# Molecular Typing and Carbapenem Resistance Mechanisms of *Pseudomonas aeruginosa* Isolated From a Chinese Burn Center From 2011 to 2016

**DOI:** 10.3389/fmicb.2018.01135

**Published:** 2018-05-29

**Authors:** Supeng Yin, Ping Chen, Bo You, Yulong Zhang, Bei Jiang, Guangtao Huang, Zichen Yang, Yu Chen, Jing Chen, Zhiqiang Yuan, Yan Zhao, Ming Li, Fuquan Hu, Yali Gong, Yizhi Peng

**Affiliations:** ^1^State Key Laboratory of Trauma, Burns and Combined Injury, Institute of Burn Research, Southwest Hospital, Third Military Medical University (Army Medical University), Chongqing, China; ^2^Number 324 Hospital, People’s Liberation Army, Chongqing, China; ^3^Number 474 Hospital, People’s Liberation Army, Ürümqi, China; ^4^Department of Microbiology, Third Military Medical University (Army Medical University), Chongqing, China

**Keywords:** *Pseudomonas aeruginosa*, MLST, carbapenem resistance, OprD, AmpC, efflux pump

## Abstract

*Pseudomonas aeruginosa* is the leading cause of infection in burn patients. The increasing carbapenem resistance of *P. aeruginosa* has become a serious challenge to clinicians. The present study investigated the molecular typing and carbapenem resistance mechanisms of 196 *P. aeruginosa* isolates from the bloodstream and wound surface of patients in our burn center over a period of 6 years. By multilocus sequence typing (MLST), a total of 58 sequence types (STs) were identified. An outbreak of ST111, a type that poses a high international risk, occurred in 2014. The isolates from wound samples of patients without bacteremia were more diverse and more susceptible to antibiotics than strains collected from the bloodstream or the wound surface of patients with bacteremia. Importantly, a large proportion of the patients with multisite infection (46.51%) were simultaneously infected by different STs in the bloodstream and wound surface. Antimicrobial susceptibility testing of these isolates revealed high levels of resistance to carbapenems, with 35.71% susceptibility to imipenem and 32.14% to meropenem. To evaluate mechanisms associated with carbapenem resistance, experiments were conducted to determine the prevalence of carbapenemase genes, detect alterations of the *oprD* porin gene, and measure expression of the *ampC* β-lactamase gene and the *mexB* multidrug efflux gene. The main mechanism associated with carbapenem resistance was mutational inactivation of *oprD* (88.65%), accompanied by overexpression of *ampC* (68.09%). In some cases, *oprD* was inactivated by insertion sequence element IS*1411*, which has not been found previously in *P. aeruginosa*. These findings may help control nosocomial *P. aeruginosa* infections and improve clinical practice.

## Introduction

*Pseudomonas aeruginosa* is one of the most common pathogens isolated from burn patients throughout the world (Revathi et al., 1998; Singh et al., 2003; Yali et al., 2014; Dou et al., 2017; Sousa et al., 2017). The increasing prevalence of multidrug-resistant (MDR), extensively drug resistant (XDR), and pandrug-resistant (PDR) *P. aeruginosa* poses a grim challenge for antimicrobial therapy (El Zowalaty et al., 2015). Especially in burn centers, the high prevalence and progressive increasing of MDR *P. aeruginosa* seriously threats the patients with severe burn injure (de Almeida Silva et al., 2017; Dou et al., 2017). Therefore, continuous surveillance of this high-risk pathogen and understanding its resistance mechanisms are important to effectively guide clinical treatment and support infection control programs, as well as to prevent its global dissemination.

Currently, carbapenems are the main antibiotics used for treating MDR *P. aeruginosa* infections. However, carbapenem resistance is increasing year by year especially in the isolates collected from burn patients (Dou et al., 2017; Sousa et al., 2017) and thus presents a difficult challenge for clinicians. Carbapenem resistance in *P. aeruginosa* is usually multifactorial and can be caused by several different mechanisms (Castanheira et al., 2014). Generally, *P. aeruginosa* can acquire resistance to carbapenems by acquisition of transferable genes encoding carbapenemases, such as the metallo-β-lactamases (MBLs), *Klebsiella pneumoniae* carbapenemases (KPC), and GES enzymes (Queenan and Bush, 2007; Tzouvelekis et al., 2012; Liakopoulos et al., 2013). Moreover, repression or inactivation of the carbapenem porin OprD and hyperexpression of the chromosomal cephalosporinase AmpC are associated with the reduced susceptibility to carbapenems (Lister et al., 2009; Cabot et al., 2011; Li et al., 2012). In addition, the overexpression of efflux pump system such as MexAB-OprM also contributes directly to meropenem resistance (Masuda et al., 1995, 2000; Lister et al., 2009). These mechanisms alone or together confer *P. aeruginosa* resistance to carbapenems.

Wound infections and bacteremia caused by *P. aeruginosa* usually happen in severe burn patients and are often refractory. In order to understand the characteristics of *P. aeruginosa* molecular typing and antimicrobial resistance profiles in our center, we collected and analyzed 196 *P. aeruginosa* strains isolated from the wound surface and bloodstream (Bl) of burn patients for a period of 6 years (2011–2016). A subset of 141 carbapenem-non-susceptible isolates was selected to evaluate their major resistance mechanisms, with emphasis on the prevalence of carbapenemase genes, upregulation of AmpC and efflux pump MexAB-OprM, and loss or alteration of OprD.

## Materials and Methods

### Ethics Statement

This study was approved by the Ethics Committee of Southwest Hospital, Third Military Medical University. No written informed consent was required because we received anonymized isolate samples with all the personal information removed.

### Bacterial Strains

Clinical *P. aeruginosa* isolates were collected from 2011 to 2016 at the Institute of Burn Research at Southwest Hospital in Chongqing, one of the largest burn center in China, with 150 beds that takes a mass of patients mainly from southwest China. Strains were isolated from the Bl and wound surface of burn patients and identified using the API 20 NE system (BioMerieux) and 16S rRNA gene sequence analysis (AbdulWahab et al., 2015). The isolates were grouped depending on whether they were obtained from the Bl, the wound surface of a patient without bacteremia (WN), or the wound surface of a patient with bacteremia (WB). Every strain was the first isolate of a series of sample collection during hospitalization that isolated from the specific source of the patient. *P. aeruginosa* PAO1 (Jacobs et al., 2003) was used as a reference strain in the quantification of *ampC* and the efflux pump gene expression.

### Multilocus Sequence Typing

Multilocus sequence typing was performed as described in the PubMLST database of *P. aeruginosa*^1^. Briefly, genomic DNA from each isolate was extracted from cultures grown to the late exponential phase by using a Genomic DNA Purification Kit (Promega). The resulted genomic DNA was used as template to amplify seven housekeeping genes (*acsA, aroE, guaA, mutL, nuoD, ppsA*, and *trpE*) by PCR as described in the PubMLST database^2^ with a few modifications. 2 × Taq premix (Takara) was used in PCR according to the recommended conditions. The amplification reaction for *aroE* required the addition of 5% DMSO (dimethyl sulfoxide). The PCR products were purified with Gel Extraction Kit (OMEGA) and then underwent bidirectional sequencing using the Applied Biosystems (ABI) 3730 DNA analyzer. Gene sequences were used to query the PubMLST database to identify matches to known (numbered) alleles. The seven allele numbers were combined to construct an identifier for a ST. The types that could not match any known types were deposited to obtain new STs. BioNumerics (version 7.6) was used to analyze the clonal relationships between the STs and create a minimum spanning tree. A clonal complex (CC) was defined to contain at least two STs sharing any six of the seven alleles.

### Antimicrobial Susceptibility Testing

Susceptibility to piperacillin, piperacillin/tazobactam, ceftazidime, cefepime, sulbactam/cefoperazone, amikacin, gentamicin, netilmicin, ciprofloxacin, and levofloxacin was tested for all isolates using the K-B agar diffusion method (CLSI 2011–2017, M100-S21-M100-S27). MICs of imipenem, meropenem, and polymyxin B were determined by the microdilution method. Susceptibility was categorized using the breakpoints defined by the Clinical and Laboratory Standards Institute guidelines (CLSI 2011–2017, M100-S21-M100-S27) and European Committee on Antimicrobial Susceptibility Testing (EUCAST, version 7.1, http://www.eucast.org, for polymyxin B only).

### Gene Amplification and Sequencing

Multiplex PCR was used to detect acquired carbapenemase genes in carbapenem-non-susceptible isolates as described previously (Ellington et al., 2007; Poirel et al., 2011). Eleven genes were divided into 3 groups as follows: group 1 *bla*_IMP_, *bla*_V IM_, *bla*_NDM_, and *bla*_GES_, group 2 *bla*_SPM_, *bla*_BIC_, and *bla*_KPC_, group 3 *bla*_AIM_, *bla*_GIM_, *bla*_SIM_, and *bla*_DIM_. One microliter of DNA (50 ng) was subjected to multiplex PCR in a 20 μL reaction mixture with 10 μmol/L of each primer shown in **Supplementary Table S1** (Ellington et al., 2007; Poirel et al., 2011) and 10 μL 2 × Taq premix (Takara). When necessary, 5% DMSO was added to the reaction. Amplified products detected by agarose gel electrophoresis were sequenced.

The full-length *oprD* gene from each isolate was amplified and sequenced using the primers in **Supplementary Table S1** (Rodriguez-Martinez et al., 2009). DNA sequences were compared with the *oprD* sequence from the reference strain PAO1 using MEGA7 (Kumar et al., 2016).

### Quantitative Real-Time PCR

Expression of *ampC, mexB*, and *oprD* was measured using quantitative real-time PCR. Total RNA was extracted using TriPure RNA isolation reagent (Roche) and reverse transcribed to cDNA with a first-strand cDNA synthesis kit (Thermo Fisher Scientific). RT-qPCR was then performed in a CFX Connect Real-Time PCR System (BIO-RAD) using SYBR green real-time PCR master mix (TOYOBO). The *rpsL* gene was used as an internal reference. Primers for all the genes in RT-qPCR are shown in **Supplementary Table S1** (Juan et al., 2006; Bubonja-Sonje et al., 2015). Three independent experiments were performed and the mean values of relative expression for each gene were compared with their corresponding expression levels in PAO1. The evaluation criteria which are used widely in many other studies were as previously described (Oh et al., 2003; Cabot et al., 2011; Vatcheva-Dobrevska et al., 2013; Bubonja-Sonje et al., 2015). For *ampC* overexpression is defined as a level at least 10-fold higher than the corresponding level in PAO1, negative if less than 5-fold higher, and borderline if between 5- and 10-fold. For *mexB*, overexpression indicates a level at least 3-fold higher, negative indicates the level is less than 2-fold, and borderline indicates the level is between 2- and 3-fold higher.

### Statistical Analysis

Data were analyzed using the GraphPad Prism analysis package. The antimicrobial susceptibility of the isolates in different groups was compared using Pearson’s chi-square test. *P* < 0.05 was considered statistically significant.

## Results

### Molecular Typing of the *P. aeruginosa* Clinical Isolates

**FIGURE 1:**
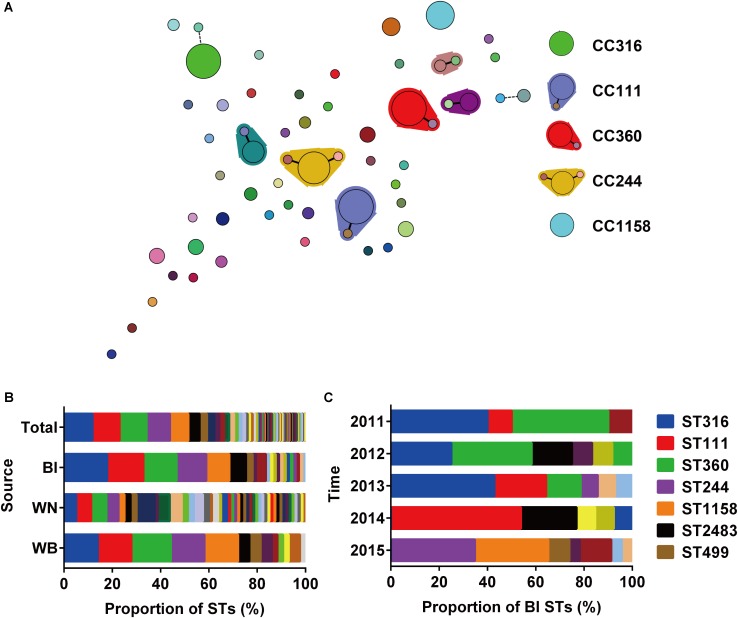
Relationships and population analysis of sequence types (STs). **(A)** Minimum spanning tree of STs created by BioNumerics. Each solid circle denotes one ST, and the area of the circle is proportional to the number of isolates. A solid or dashed line between circles indicates that the two linked STs share six or five identical alleles. STs enclosed by shaded areas constitute a CC. The main STs and CCs are shown on the right. **(B)** Composition of STs isolated from different sources. Bl, bloodstream; WN, wound samples from patients without bacteremia; WB, wound samples from patients accompanied by bacteremia. **(C)** Composition of STs among strains isolated from the bloodstream at different times.

A total of 196 isolates from different sources (Bl = 73, WN = 80, WB = 43) were analyzed by MLST to investigate their clonal relationships. The results revealed high clonal diversity, with 42 known STs identified among 171 isolates, and 16 new STs among 25 isolates (**Supplementary Table S2**). ST316, ST111, ST360, ST244, and ST1158 were the dominant STs with at least 15 isolates in each type, and accounted for 51.53% of the total isolates. Six CCs were identified, in which CC111, CC360, and CC244 were considered as large CCs (**Figure 1A**). The top 5 STs accounted for 68.49 or 72.09% of the isolates collected from the Bl or wound surface of patients WB, respectively. However, among strains isolated from wound samples of patients WN, only 25 percent belonged to these 5 STs (**Figure 1B**). This result indicates that *P. aeruginosa* isolated from WN were more diverse than those isolated from Bl or WB. Among isolates from Bl, the main STs varied depending on the year of collection. In 2011 and 2012, ST316 and ST360 were the dominant types. Beginning in 2013, ST111 emerged and became the primary type in 2014 along with ST2483. In 2015, both lost their position to ST244 and ST1158 (**Figure 1C**). As there was only one isolate from Bl in 2016 which belonged to ST270, it was not presented in **Figure 1C**. In June and July 2014, 6 strains of ST111 caused an outbreak of Bl infections in our center (**Supplementary Table S3**).

Notably, in the 43 patients WB accompanied by wound infections, only 23 patients were infected with identical STs in their Bl and wound surface, while the other 20 patients were infected with two different STs simultaneously (**Table 1**).

**Table 1:** STs of isolates collected from the bloodstream and wound surface of patients with bacteremia accompanied by wound infection.

Patient	Bl	WB	Patient	Bl	WB	Patient	Bl	WB
**1**^a^	ST360	ST316	**2**	ST316	ST360	3	ST360	ST360
**4**	ST111	ST316	**5**	ST623	ST360	6	ST360	ST360
7	ST360	ST360	**8**	ST360	ST316	9	ST316	ST316
**10**	ST316	ST111	**11**	ST316	ST360	12	ST2483	ST2483
**13**	ST2494	ST360	**14**	ST316	ST111	**15**	ST360	ST365
16	ST316	ST316	**17**	ST316	ST111	18	ST111	ST111
**19**	ST111	ST316	**20**	ST2483	ST111	21	ST2483	ST2483
22	ST111	ST111	**23**	ST782	ST2492	24	ST2488	ST2488
25	ST499	ST499	26	ST499	ST499	27	ST244	ST244
28	ST244	ST244	29	ST385	ST385	30	ST1158	ST1158
31	ST1158	ST1158	**32**	ST385	ST1158	**33**	ST1158	ST244
**34**	ST244	ST1158	35	ST244	ST244	36	ST260	ST260
37	ST1158	ST1158	38	ST244	ST244	**39**	ST1158	ST245
40	ST244	ST244	**41**	ST244	ST260	42	ST1158	ST1158
**43**	ST2481	ST2493						

Bl, samples from bloodstream; WB, wound samples from patients accompanied by bacteremia. ^a^Numbers in boldface indicate patients who were infected simultaneously by two different STs of P. aeruginosa in the bloodstream and wound.

### Antimicrobial Susceptibility Profiles

The susceptibility test (raw data are shown in **Supplementary File 1**) revealed that 107 (54.59%) of the isolates exhibited an MDR phenotype. 42 (21.42%) strains were XDR and sensitive only to polymyxin B, which is rarely used in China. More seriously, these isolates showed high resistance to carbapenems, with only 70 (35.71%) of them susceptible to imipenem and 63 (32.14%) to meropenem. Since carbapenems are currently the most important therapeutic option to treat infections caused by MDR *P. aeruginosa*, the 141 carbapenem-non-susceptible isolates (either non-susceptible to imipenem or meropenem) were selected for further study to investigate their resistance mechanisms.

**FIGURE 2:**
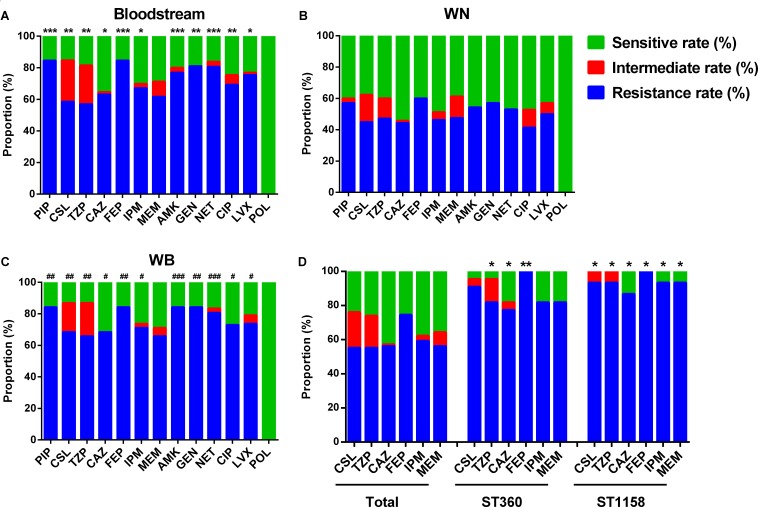
Antimicrobial susceptibility of differently sourced isolates. PIP, Piperacillin; CSL, sulbactam/cefoperazone; TZP, piperacillin/tazobactam; CAZ, ceftazidime; FEP, cefepime; IPM, imipenem; MEM, meropenem; AMK, amikacin; GEN, gentamicin; NET, netilmicin; CIP, ciprofloxacin; LVX, levofloxacin; POL, polymyxin B. **(A)** Isolates from bloodstream; **(B)** WN, isolates from wound samples of patients without bacteremia; **(C)** WB, isolates from wound samples of patients accompanied by bacteremia. ^∗^*P*< 0.05, ^∗∗^*P*< 0.01, and ^∗∗∗^*P*< 0.001 indicate differences are significant between Bl and WN; ^#^*P*< 0.05, ^##^*P*< 0.01, and ^###^*P*< 0.001 indicate differences are significant between WB and WN. **(D)** ^∗^*P*< 0.05, ^∗∗^*P*< 0.01 compared with that of the total isolates.

Compared with isolates collected from WN, the samples from Bl and WB showed higher resistance rate to the vast majority of the antibiotics tested including piperacillin (PIP), sulbactam/cefoperazone (CSL), piperacillin/tazobactam (TZP), ceftazidime (CAZ), cefepime (FEP), imipenem (IPM), amikacin (AMK), gentamicin (GEN), netilmicin (NET), ciprofloxacin (CIP), and levofloxacin (LVX) (**Figures 2A–C**). This suggests that *P. aeruginosa* isolated from the patients WB is more resistant than that isolated from patients with only wound infections.

In the top 5 STs, although no ST was associated with any specific resistance profile, ST360 and ST1158 showed higher resistance rate to most of the commonly used antibiotics in burn centers such as sulbactam/cefoperazone (90.91 and 93.33%), piperacillin/tazobactam (81.28 and 93.33%), ceftazidime (77.27 and 86.67%), cefepime (100 and 100%), imipenem (81.82 and 93.33%), and meropenem (81.82 and 93.33%) (**Figure 2D**).

### Presence of Carbapenemase Genes

Of the 141 carbapenem-non-susceptible *P. aeruginosa*, 11 (7.80%) isolates were positive for *bla*_IMP_ and 9 (6.38%) isolates harbored *bla*_V IM_. Sequence analysis showed that these two genes encode IMP-9 and VIM-2, respectively (**Table 2**). No other carbapenemase genes were detected by PCR screening. Among these carbapenemase-positive isolates, ST499 (6 isolates) and ST111 (5 isolates) were the main types. One ST499 strain was positive for both *bla*_IMP_ and *bla*_V IM_. Among the strains producing IMP-9, apart from 6 strains of ST499 and 2 strains of ST111, there was only 1 strain of ST244, ST316, and ST1028, respectively. While, in the strains harboring VIM-2, there were 3 strains of ST111 and ST260, respectively, 2 of ST1158, and only 1 of ST499. None of these STs that producing IMP-9 or VIM-2 belonged to the same CC.

**Table 2:** Prevalence of acquired carbapenemase-encoding genes and *ampC* or efflux pump *mexB* overexpression in carbapenem-non-susceptible isolates.

Resistance genes		No. (%) of isolates	


	Overexpression^a^	Borderline	No overexpression
*ampC*	96 (68.09)	12 (8.51)	33 (23.40)
*mexB^b^*	17 (12.78)	20 (15.04)	96 (72.18)
IMP-9	11 (7.80)		
VIM-2	9 (6.38)		

^a^The mRNA levels of the indicated genes in PAO1 were used as controls. Evaluation criteria are as follows: for ampC overexpression indicates expression at least 10-fold higher than in PAO1, negative indicates expression is lower than 5-fold, and borderline indicates expression is between 5- and 10-fold higher. For mexB, overexpression indicates expression at least 3-fold higher, negative indicates expression is less than 2-fold higher, and borderline indicates expression is between 2- and 3-fold higher. ^b^Transcription of mexB was determined in 133 meropenem-non-susceptible isolates.

### Expression of *ampC* and *mexB*

Expression levels for *ampC* β-lactamase gene and the *mexB* multidrug efflux gene among carbapenem-non-susceptible or meropenem-non-susceptible isolates are shown in **Table 2**. AmpC hyperproduction is most common in these isolates. Ninety-six (68.09%) isolates were found to meet criteria for overexpression, and 12 (8.51%) isolates showed a borderline increase. The efflux pump gene *mexB* was overexpressed in 12.78% of the meropenem-non-susceptible isolates.

### Mutational Inactivation and Expression of OprD Porin

Mutational inactivation of the *oprD* gene is the major reason for OprD loss in *P. aeruginosa* (Rodriguez-Martinez et al., 2009; Fang et al., 2014). The *oprD* gene sequence was therefore determined in all 141 carbapenem-non-susceptible isolates (**Table 3**). 125 (88.65%) isolates were found to have various inactivating mutations, potentially resulting in truncated or aberrant proteins. 95 strains contained frameshift mutations due to deletions or insertions of 1 or several base pairs. Premature stop codons caused by point mutations were detected in 12 isolates. In addition, two insertion sequence (IS) elements were found to have interrupted *oprD*. In 13 ST1158 isolates, IS*1411* inserted into the coding sequence of *oprD*, while in 5 ST499 isolates, IS*1394* inserted upstream of the start codon in a location that could influence transcription initiation or translation. In the 16 isolates without inactivating mutations, 8 contained amino acid substitutions and no mutations were identified in 6 strains. In the 8 strains containing amino acid substitutions, 5 of them were resistant to imipenem, and the other 3 were sensitive. While in meropenem susceptibility assay, 5 were resistant, 2 were intermediate, and 1 was sensitive. This result suggests that there is no necessary connection between these substitutions and carbapenem resistance. Two isolates were negative by PCR detection. We speculate that these may have suffered more serious changes affecting the *oprD* gene, such as large deletions (Fang et al., 2014). We further measured *oprD* expression in these 14 isolates without inactivating mutations, and all of them showed reduced *oprD* expression (**Supplementary Table S4**).

**Table 3:** Mutations inactivating *oprD* in carbapenem-non-susceptible isolates.

Type of mutation	Mutational characteristics*^a^*	STs (no. of isolates)
Frameshift mutation	1-bp deletion (G) at nt 276	ST360 (19)
	1-bp deletion (T) at nt 912	ST111 (22)
	1-bp deletion (A) at nt 886	ST385 (1)
	1-bp deletion (G) at nt 376	ST782 (1)
	1-bp deletion (T) at nt 667	ST385 (3)
	2-bp deletion (AT) at nt 1114-1115	ST316 (11), ST260 (4), ST2483 (4), ST485 (3), ST980 (2), ST274 (2), ST170 (1), ST270 (1), ST365 (1), ST499 (1), ST633 (1), ST782 (1), ST1028 (1), ST2420 (1), ST2492 (1), ST2493 (1), ST2494 (1)
	2-bp deletion (TG) at nt 2-3	ST2479 (1)
	10-bp deletion at nt 126-143 (**C**GA**CCT**GC**TG**CT**CC**GC**AA**)	ST385 (1)
	10-bp deletion at nt 858-874 (**TG**CGC**A**C**A**C**TTT**C**A**C**CT**)	ST292 (1)
	5-bp insertion (ATGGC) at nt 1054-1055	ST244 (4)
	5-bp insertion (GGCCG) at nt 925-926	ST244 (3), ST595 (1)
	7-bp insertion (CCTGTTC) at nt 469-470	ST244 (1)
Premature stop codon	**C**A**A**→**T**A**G** at nt 199-201	ST316 (4)
	**G**AA→**T**AA at nt 220-222	ST316 (3)
	T**G**G→T**A**G at nt 16-18	ST639 (1)
	TG**G**→TG**A** at nt 1015-1017	ST244 (2), ST385 (1)
	**C**AG→**T**AG at nt 496-498	ST244 (1)
IS insertion	IS*1411* beginning at nt 788	ST1158 (13)
	IS*1394* beginning at nt -11	ST499 (4)
	IS*1394* beginning at nt -1	ST499 (1)
Amino acid substitution	T103S, K115T, F170L	ST277 (3), ST2488 (2), ST2478 (1)
	T103S, K115T, F170L, E185Q, P186G, V189T, R310E, A315G, G425A	ST245 (1), ST1648 (1)
No mutation	None	ST244 (2), ST554 (3), ST111 (1)
Negative by PCR	Unknown change	ST1158 (2)

IS, insertion sequence; nt, nucleotide. ^a^Sequences of oprD were compared to oprD in the reference strain PAO1. Bases in boldface indicate mutated nucleotides.

## Discussion

*Pseudomonas aeruginosa* is a major pathogen that often causes nosocomial infections in burn patients (Revathi et al., 1998; Singh et al., 2003; Yali et al., 2014; Sousa et al., 2017). The rapid increase of multidrug resistance and even carbapenem resistance exhibited by this bacterium makes it a serious problem in burn centers (Dou et al., 2017; Sousa et al., 2017). Our previous study showed that *P. aeruginosa* is consistently one of the top three microorganisms isolated in our center (Yali et al., 2014).

A high clonal diversity of *P. aeruginosa* was identified in our center during a 6 year period. ST316, ST111, ST360, ST244, and ST1158 were the major STs or CCs. Among the STs, ST316, and ST244 are in the top 10 major types in China (Ji et al., 2013). ST360 is also a major ST in a burn center in Iran (Fazeli et al., 2014) and showed a high rate of resistance in the present study. ST111, one of the clones that pose a high international risk, usually produces carbapenemases (Oliver et al., 2015). We observed that it caused an outbreak of Bl infections in our center in June and July 2014 (**Supplementary Table S3**). Most of these isolates were XDR and sensitive only to Polymyxin B. ST111 isolates were also found to have a high prevalence to produce IMP-9 and VIM-2, thus may be responsible for the spread of carbapenem resistance in our center. Although ST1158 was not widespread, a majority of these isolates (13/15) harbored IS*1411* in the *oprD* gene, which may explain its higher resistance to carbapenems and some other commonly used antibiotics in the clinic. In consideration of its current prevalence and high antimicrobial resistance, it emphatically needs to be monitored in the future.

The constitution of the major STs and their antimicrobial susceptibility profiles also vary according to sample source. Although strains isolated from wound samples of patients (WN were more diverse and complex than those collected from patients with bacteremia (Bl and WB), they were less resistant to most of the antibiotics tested. This suggests that clinicians treating wound infections should differentiate between WN and WB because of the different drug resistance spectra offered by the infecting strains.

It was at one time assumed that if a particular bacterial species were obtained from different sources in the same patient, the isolates would be identical strains. In order to avoid duplicates from the same isolates, the first strain isolated from a patient was usually selected and studied. However, in 43 patients WB accompanied by wound infections, 46.51% of the patients were found to be infected by different STs in the Bl and wound surface. This suggests that the bacteria in the Bl of burn patients not only originate from infected burn wounds, but may also come from sources such as intestinal flora, or be the result of nosocomial infections (Church et al., 2006). Moreover, different strains might have different antimicrobial susceptibility profiles. This should also be taken into consideration in the treatment of burn patients, especially in the choice of antibiotics.

Carbapenems are usually the first choice for the treatment of MDR *P. aeruginosa* infections in burn centers. Resistance to these drugs severely hampers their efficacy. Carbapenem resistance mechanisms are multifactorial. In the present study, the presence of carbapenemase genes seems less common in carbapenem-non-susceptible isolates than is the case in some countries (Wolter et al., 2009; Liakopoulos et al., 2013; Castanheira et al., 2014). The main resistance mechanism was mutational inactivation of OprD porin, accompanied by hyperproduction of AmpC, representing 68.09% isolates. Such high rate of AmpC hyperproduction is inconsistent with another study reported in China which found only 5.4% (14/258) of the carbapenem-resistant isolates to overexpress *ampC* (Wang et al., 2010). The upregulation of MexAB-OprM efflux system may increase the resistance to meropenem (Kohler et al., 1999; Chalhoub et al., 2016). However, only 12.78% of the meropenem-non-susceptible isolates showed an overexpression of *mexB*. Thus, our data demonstrate that mutations in *oprD*, accompanied by overexpression of AmpC, are even more often combined to produce resistance. Compared with the high prevalence of carbapenemase genes in some European nations, these intrinsic resistance mechanisms in a highly genetically diverse population of carbapenem-non-susceptible *P. aeruginosa* are probably a matter of greater concern in China.

Mutational inactivation of OprD is primarily responsible for the resistance to imipenem, and is reported to be the most common mechanism in China and Korea (Fang et al., 2014; Kim et al., 2016). Frameshifts, caused by deletions or insertions of 1 or more base pairs, are the most frequent type of mutation (Rodriguez-Martinez et al., 2009; Yan et al., 2014). The insertion of two different IS elements was also common (18 isolates). IS*1394* was previously reported as an insertion in the coding sequence of *oprD* or 1-bp upstream of the start codon (Wolter et al., 2009; Wolkowicz et al., 2016). We also found it located 11 bp upstream of the coding sequence in one isolate. However, to our knowledge, ours is the first report that IS*1411* is found in *P. aeruginosa* and is responsible for a mutation in *oprD*. It was first identified in *Pseudomonas putida*, where it is associated with the transcriptional activation of phenol degradation genes (Kallastu et al., 1998). IS*1411* resembles IS*Ppu21*, another *P. putida* IS that also occurs in *P. aeruginosa* (Sun and Dennis, 2009; Rojo-Bezares et al., 2014). IS*1411* may contribute to the increasing drug resistance in *P. aeruginosa* through horizontal gene transfer.

Interestingly, the amino acid substitutions (T103S, K115T, F170L, E185Q, P186G, V189T, R310E, A315G, and G425A) detected in our study are also reported in other parts of China, Korea, Spain and France (Rodriguez-Martinez et al., 2009; Wang et al., 2010; Ocampo-Sosa et al., 2012; Rojo-Bezares et al., 2014; Kim et al., 2016). A majority of these mutations are located in the external loops of OprD that are responsible for the binding of carbapenems, but the drug resistance profiles of these isolates shows that there is no necessary connection between these substitutions and carbapenem resistance. This result is consistent with previous studies (Wang et al., 2010; Rojo-Bezares et al., 2014). However, a recent study demonstrates that the common polymorphism at codon 170 (F107L), is associated with reduced *oprD* expression and the potential to develop carbapenem resistance in PAO1 (Shu et al., 2017). This most common mutation of F107L was also found in all of the 8 isolates with amino acid substitutions in our study. And the expression of *oprD* in these isolates was also decreased. These results suggest that in clinical isolates, these frequent combined amino acid substitutions in *oprD* may be associated with carbapenem resistance, but will not inevitably lead to it. This discrepancy may attribute to the difference between standard strain PAO1 and clinical isolates. As it is more complicated in the clinical isolates, there may be some other factors rather than OprD alteration that lead to carbapenem resistance. But these factors and the effect of each single amino acid substitution in these clinical isolates need to be further studied.

## Conclusion

In conclusion, molecular epidemiological investigation of clinically isolated *P. aeruginosa* reveals that an outbreak of ST111, a clone that poses a high international risk, occurred in our center in 2014. The different genetic relatedness and antimicrobial susceptibility between the isolates from Bl and wound surface are noteworthy. Mutational inactivation of *oprD*, accompanied by the overexpression of AmpC, is the main mechanisms of carbapenem resistance. Additionally, IS*1411* was found for the first time in *P. aeruginosa* and was one of the many genetic events responsible for the inactivation of *oprD*. These results may help improve infection control measures and clinical treatment.

## Author Contributions

YP, YG, and SY conceived and designed this study. PC, SY, YLZ, BJ, GH, BY, ZCY, YC, and YG performed the experiments. PC, SY, JC, ZQY, YZ, ML, and FH analyzed the data. YP and SY drafted the manuscript.

## Conflict of Interest Statement

The authors declare that the research was conducted in the absence of any commercial or financial relationships that could be construed as a potential conflict of interest.

## Footnotes

1 http://pubmlst.org/paeruginosa/

2 https://pubmlst.org/paeruginosa/info/primers.shtml
