# Supplementary material for: Molecular Typing and Carbapenem Resistance Mechanisms of Pseudomonas aeruginosa Isolated From a Chinese Burn Center From 2011 to 2016
Source: Front Microbiol. 2018 May 29;9:1135. doi: 10.3389/fmicb.2018.01135 (PMC5987737; doi:10.3389/fmicb.2018.01135)
Supplement: Supplementary file 1 [file Table_1.docx]

***Supplementary Material***

**Molecular Epidemiology and** **Carbapenem Resistance Mechanisms of** ***Pseudomonas aeruginosa* Isolated from a Chinese Burn Center from 2011 to 2016**

Supeng Yin^1^, Ping Chen^1^, Bo You^1^, Yulong Zhang^1, 3^, Bei Jiang^1^, Guangtao Huang^1^, Zichen Yang^1^, Yu Chen^1^, Jing Chen^1^, Zhiqiang Yuan^1^, Yan Zhao^2^, Ming Li^2^, Fuquan Hu^2^, Yali Gong^1*^ and Yizhi Peng^1*^

***Corresponding authors:** Yizhi Peng. E-mail: [yizhipen@sina.com](mailto:yizhipen@sina.com)**,** Yali Gong. E-mail: gyl0804@163.com

**Table S1.** **Primers used in this study**

| **Primers** | **Sequence (5’-3’)** | **Use** |
| --- | --- | --- |
| *bla*_IMP_-F | GGAATAGAGTGGCTTAAYTCTC | Amplification of carbapenemase gene |
| *bla*_IMP_-R | GGTTTAAYAAAACAACCACC | Amplification of carbapenemase gene |
| *bla*_VIM_-F | GATGGTGTTTGGTCGCATA | Amplification of carbapenemase gene |
| *bla*_VIM_-R | CGAATGCGCAGCACCAG | Amplification of carbapenemase gene |
| *bla*_NDM_-F | GGTTTGGCGATCTGGTTTTC | Amplification of carbapenemase gene |
| *bla*_NDM_-R | CGGAATGGCTCATCACGATC | Amplification of carbapenemase gene |
| *bla*_GES_-F | ATGCGCTTCATTCACGCAC | Amplification of carbapenemase gene |
| *bla*_GES_-R | CTATTTGTCCGTGCTCAGG | Amplification of carbapenemase gene |
| *bla*_SPM_-F | AAAATCTGGGTACGCAAACG | Amplification of carbapenemase gene |
| *bla*_SPM_-R | ACATTATCCGCTGGAACAGG | Amplification of carbapenemase gene |
| *bla*_BIC_-F | TATGCAGCTCCTTTAAGGGC | Amplification of carbapenemase gene |
| *bla*_BIC_-R | TCATTGGCGGTGCCGTACAC | Amplification of carbapenemase gene |
| *bla*_KPC_-F | CGTCTAGTTCTGCTGTCTTG | Amplification of carbapenemase gene |
| *bla*_KPC_-R | CTTGTCATCCTTGTTAGGCG | Amplification of carbapenemase gene |
| *bla*_AIM_-F | CTGAAGGTGTACGGAAACAC | Amplification of carbapenemase gene |
| *bla*_AIM_-R | GTTCGGCCACCTCGAATTG | Amplification of carbapenemase gene |
| *bla*_GIM_-F | TCGACACACCTTGGTCTGAA | Amplification of carbapenemase gene |
| *bla*_GIM_-R | AACTTCCAACTTTGCCATGC | Amplification of carbapenemase gene |
| *bla*_SIM_-F | TACAAGGGATTCGGCATCG | Amplification of carbapenemase gene |
| *bla*_SIM_-R | TAATGGCCTGTTCCCATGTG | Amplification of carbapenemase gene |
| *bla*_DIM_-F | GCTTGTCTTCGCTTGCTAACG | Amplification of carbapenemase gene |
| *bla*_DIM_-R | CGTTCGGCTGGATTGATTTG | Amplification of carbapenemase gene |
| *oprD*-F | CGCCGACAAGAAGAACTAGC | Amplification and sequencing |
| *oprD*-R | GTCGATTACAGGATCGACAG | Amplification |
| *oprD*-F2 | GCCGACCACCGTCAAATCG | Sequencing |
| *ampC*-F | GGGCTGGCCTCGAAAGAGGAC | Quantitative real-time PCR |
| *ampC*-R | GCACCGAGTCGGGGAACTGCA | Quantitative real-time PCR |
| *mexB*-F | CAAGGGCGTCGGTGACTTCCAG | Quantitative real-time PCR |
| *mexB*-R | ACCTGGGAACCGTCGGGATTGA | Quantitative real-time PCR |
| *oprD-*F3 | GCTCGACCTCGAGGCAGGCCA | Quantitative real-time PCR |
| *oprD*-R3 | CCAGCGATTGGTCGGATGCCA | Quantitative real-time PCR |
| *rpsL*-F | GCTGCAAAACTGCCCGCAACG | Quantitative real-time PCR |
| *rpsL*-R | ACCCGAGGTGTCCAGCGAACC | Quantitative real-time PCR |

**Table S2.** New STs found in this study

| ST | No. of isolates | Source |
| --- | --- | --- |
| ST2420 | 1 | Bloodstream |
| ST2478 | 1 | Wound |
| ST2479 | 1 | Wound |
| ST2481 | 1 | Bloodstream |
| ST2483 | 9 | Bloodstream, Wound |
| ST2484 | 1 | Wound |
| ST2485 | 1 | Wound |
| ST2486 | 1 | Wound |
| ST2488 | 2 | Bloodstream, Wound |
| ST2489 | 1 | Wound |
| ST2490 | 1 | Wound |
| ST2492 | 1 | Wound |
| ST2493 | 1 | Wound |
| ST2494 | 1 | Bloodstream |
| ST2495 | 1 | Bloodstream |
| ST2496 | 1 | Wound |

**Table S3.** Time course of the ST111 isolates collected in June and July 2014

| Patient | Strain | Source | Time |
| --- | --- | --- | --- |
| 1 | B42 | Bloodstream | 2014-06-06 |
| 2 | B43 | Bloodstream | 2014-06-07 |
| 3 | B44 | Bloodstream | 2014-06-08 |
| 4 | B45 | Bloodstream | 2014-06-10 |
| 5 | B48 | Bloodstream | 2014-07-26 |
| 6 | B50 | Bloodstream | 2014-07-31 |

**Table S4.** *oprD* expression of the isolates without inactivating mutations in *oprD*

| Strain | Fold change of *oprD* relative to PAO1 |
| --- | --- |
| Strains with Amino acid substitution |  |
| B49 | 0.15 |
| W4 | 0.001 |
| W28 | 0.57 |
| W39 | 0.46 |
| W41 | 0.02 |
| W53 | 0.45 |
| W79 | 0.21 |
| WB34 | 0.32 |
| Strains without mutation |  |
| B18 | 0.01 |
| W34 | 0.13 |
| W35 | 0.05 |
| W40 | 0.18 |
| WB37 | 0.22 |
| WB39 | 0.001 |

**Supplementary File 1.** Raw data of the susceptibility test (Sheet 1_v1.XLSX).
